# MiR-143-3p Inhibits Osteogenic Differentiation of Human Periodontal Ligament Cells by Targeting KLF5 and Inactivating the Wnt/β-Catenin Pathway

**DOI:** 10.3389/fphys.2020.606967

**Published:** 2021-02-02

**Authors:** Kaixin Wangzhou, Zhiying Lai, Zishao Lu, Wanren Fu, Cheng Liu, Zhengeng Liang, Yi Tan, Conghui Li, Chunbo Hao

**Affiliations:** ^1^School of Management, Hainan Medical University, Haikou, China; ^2^College of Stomatology, Hainan Medical University, Haikou, China; ^3^Department of Stomatology, Harbin Stomatological Hospital, Harbin, China; ^4^Department of Stomatology, Hainan General Hospital, Hainan Affiliated Hospital of Hainan Medical University, Haikou, China

**Keywords:** human periodontal ligament cells, osteogenic differentiation, microRNA-143-3p, KLF5, Wnt/β-catenin pathway

## Abstract

Human periodontal ligament cells (hPDLCs) play a vital role in cell regeneration and tissue repair with multi-directional differentiation potential. microRNAs (miRs) are implicated in the osteogenesis of hPDLCs. This study explored the mechanism of miR-143-3p in osteogenesis of hPDLCs. Osteogenic differentiation of isolated hPDLCs was induced. KLF5 expression during osteogenic differentiation of hPDLCs was detected and then silenced in hPDLCs. Binding relationship between KLF5 and miR-143-3p was predicted and verified. hPDLCs were treated with miR-143-3p mimic or overexpressing KLF5, and then osteogenic specific markers and mineralized nodules were measured. The key factors of the Wnt/β-catenin pathway during osteogenesis of hPDLCs were measured. KLF5 expression was upregulated during osteogenesis of hPDLCs. KLF5 silencing or miR-143-3p mimic reduced osteogenic specific markers and mineralized nodules. Overexpression of KLF5 could reverse the inhibitory effect of miR-143-3p on osteogenic differentiation. miR-143-3p mimic and KLF5 silencing inactivated the Wnt/β-catenin pathway. Activation of the Wnt/β-catenin pathway reversed the repression effect of miR-143-3p mimic on osteogenesis of hPDLCs. In conclusion, miR-143-3p inhibited osteogenic differentiation of hPDLCs by targeting KLF5 and inactivating the Wnt/β-catenin pathway.

## Introduction

Periodontal ligament (PDL) tissue plays a crucial role in tooth preservation by structurally maintaining the connection between the tooth root and the bone (Zhang et al., 2018). Human PDL cells (hPDLCs) are heterogeneous cells sharing the features of mesenchymal stem cells (MSCs) and viewed as promising stem cells for periodontal regeneration therapy (Tomokiyo et al., 2019; Trubiani et al., 2019). hPDLCs can differentiate into osteoblasts, adipocytes, and cementoblasts, contributing to the physiological healing of cementum-periodontal ligament complex and alveolar bone (Son et al., 2019). Osteogenic differentiation enables hPDLCs to be a potential target for the promotion of functional restoration of periodontal tissues (Kato et al., 2011). Therefore, elucidating the mechanism of osteogenesis of hPDLCs has practical significance for periodontal regeneration and osteogenic tissue engineering.

Krüppel-like factors (KLFs) are a class of transcription factors that can mediate cell proliferation, apoptosis, and differentiation (Chen et al., 2017). KLF5 has three cysteine-2/histidine-2 zinc finger motifs, which can modulate biological processes such as embryonic development, cell proliferation, and differentiation (Diakiw et al., 2013). KLF5 participates in the cell proliferation during tooth development and also relates to the mineralization of enamel and dentin matrix (Chen et al., 2009). KLF5 can enhance the differentiation of odontoblasts and promote the mineral formation of dental papilla mesenchymal cells in a mouse model (Chen et al., 2017). At present, the function of KLF5 in the osteogenesis of hPDLCs is still an intriguing issue waiting to be explored.

microRNAs (miRs), a highly conserved endogenous nonprotein RNAs, modulate gene expression post-transcriptionally *via* binding to the 3' untranslated region of target mRNAs (Wang et al., 2019b). Particularly, miRs have been demonstrated to participate in the modulation of various cellular behaviors, including differentiation, metabolism, proliferation, apoptosis, and viral infection (Huang et al., 2011). Frith et al. (2018) have suggested a novel application of mechanosensitive miRs in harnessing mechanical signaling to induce MSC differentiation towards osteogenesis in soft hydrogels. miRs also play an indispensable role in the differentiation of hPDLCs exposed to different osteogenic conditions by targeting osteogenic markers or osteogenic related pathways (Wen et al., 2020). Jia et al. (2018) have determined miR-143-3p as a potential novel biomarker for the osteogenesis of adipose-derived stem cells by using the hub nodes and the number of relationship pairs. miR-143-3p is implicated in the repression of osteogenic differentiation of bone marrow mesenchymal stem cells (BMSCs) by targeting ARL6 and downregulating the Wnt/β-catenin pathway (Wu et al., 2020). However, whether the miR-143-3p/KLF5 axis has influences on the osteogenesis of hPDLCs remains unclear. This study herein investigated the role and mechanism of miR-143-3p/KLF5 in the osteogenesis of hPDLCs, which shall provide a new theoretical basis for the management of periodontal diseases.

## Materials and Methods

### hPDLC Culture

The experimental samples were collected from six volunteers (aged 13–18 years) in Hainan General Hospital, including three females and three males. The usage of teeth was approved by the patients or their guardians. Complete, caries- and periodontitis-free premolars or third molars were extracted from the six patients due to impaction or orthodontics. The root surface was repeatedly rinsed with phosphate-buffered saline (PBS) containing 1% penicillin and streptomycin (HyClone Company, Logan, UT, USA) until the foreign matters were removed and the root became white. One third of the periodontal ligament tissues in the root was scraped off with a surgical blade and then placed in the detachment solution (Sigma-Aldrich, Merck KGaA, Darmstadt, Germany) containing 3 g/L collagenase I and 4 g/L dispase at 37°C for 1 h. Next, the detached cells and tissue blocks were collected by centrifugation at 1000 g for 5 min and cultured in the α-modified minimum Eagle’s medium (α-MEM, HyClone) supplemented with 20% fetal bovine serum (FBS; HyClone), 100 U/ml penicillin and 100 mg/L streptomycin. Afterwards, the cells were resuspended and cultured in a T25 cell culture bottle (Falcon, Munich, Germany) at 37°C with 5% CO_2._ The medium was refreshed every 3 days. The cells were detached with 0.25% trypsase (HyClone) and passaged when reaching sub-confluence. hPDLCs at passage 3 were used for the subsequent experiments.

### Identification of PDLCs

The surface markers of hPDLCs were characterized using flow cytometry. Briefly, hPDLCs were washed with PBS, treated with EDTA-trypsin for 2 min and cultured in the medium containing serum. Then, the samples were centrifuged at 100 *g* for 5 min to remove the supernatant. The precipitate was resuspended in cold PBS, centrifuged at 100 *g* for 5 min, and repeated three times. The samples were labeled with CD34 (ab81289, Abcam Inc., Cambridge, MA, USA), CD45 (ab40763, Abcam), CD73 (ab133582, Abcam), and CD105 (ab231774, Abcam) at 4°C in the dark. The cells were incubated with homologous antibody (ab6854, Abcam), and the non-specific fluorescence was measured. After 20-min incubation, the cells were washed with cold PBS containing 1% bovine serum albumin and analyzed by a flow cytometer (Beckman Coulter, Fullerton, CA, USA).

hPDLCs were seeded into 24-well plates (4 × 10^4^ cells/well). Immunohistochemistry was conducted when the cell confluence reached 60%. Each well was added with 500 μl of 4% paraformaldehyde (C104190, Aladdin, Shanghai, China), and the cells were cultured for 30 min. Following washing with PBS twice, the cells were incubated with 0.2% Triton X-100 for 30 min and 3% hydrogen peroxide for 30 min. After washing with PBS, the cells were cultured with anti-vimentin [#5741, Cell Signaling Technology (CST), Beverly, MA, USA] at 37°C for 30 min (Bian and Xiang, 2020) and treated with two drops of regent 1 for 20 min and two drops of regent 2 for 30 min. Thereafter, the cells were developed with 100–400 μl of 2,4-diaminobutyric acid and counterstained with hematoxylin (#14166, CST) for 10 min, followed by observation under a microscope (Nikon, Tokyo, Japan).

### hPDLC Transfection and Grouping

KLF5 siRNA and its NC were purchased from GenePharma (Shanghai, China). pcDNA-KLF5 and pcDNA-NC were purchased from Sangon Biotech (Shanghai, China). miR-143-3p mimic and its NC were purchased from RiboBio (Guangzhou, Guangdong, China). The well-grown hPDLCs were seeded into the 24-well plates (4 × 10^4^ cells/well), and incubated in growth medium for 24 h to make the cells reach 60–70% confluence. Then, the cells were transfected (siRNA: 40 nM; miRNA mimic: 50 nM; pcDNA: 40 nM) using the Lipofectamine 2000 (Invitrogen Inc., Carlsbad, CA, USA). hPDLCs were treated with Wnt pathway activator 2 (IC_50_: 28–29 nM; MedChemExpress, Monmouth Junction, NJ, USA) or PBS. The osteogenic induction of hPDLCs in each group was performed.

### Osteogenic Induction of hPDLCs

hPDLCs were seeded into the 24-well plates (4 × 10^4^ cells/well). Upon reaching 60–70% confluence, the cells were incubated in α-MEM containing 10% FBS, 0.1 mM dexamethasone, 0.2 mM ascorbic acid and 10 mM β-glycerophosphate (Sigma-Aldrich) continuously for 7, 14, and 21 days. The medium was refreshed every 3 days. Alizarin red S (ARS) staining and alkaline phosphatase (ALP) staining were performed on the 7th, 14th, and 21st day.

### ALP Staining

After osteogenic induction of hPDLCs, the original culture medium was removed. hPDLCs were washed with PBS, covered with appropriate amount of BCIP/NBT dyeing solution, cultured at room temperature in the dark for 5–30 min, and observed under the light microscope. Each well was added with 400 μl of cell lysate. After 30-min incubation at 4°C, the lysate was transferred into the EP tube and centrifuged at 1000 *g* for 10 min to collect the supernatant. Afterwards, each well was added with 100 μl of reaction termination solution. The operations were carried out in strict accordance with the instructions of ALP test kit (Thermo Fisher Scientific Inc., Waltham, MA, USA). The absorbance at 405 nm was determined on the microplate reader (BioTek, Winooski, VT, USA).

### ARS Staining and Analysis

After osteogenic induction of hPDLCs, the original culture medium was removed. hPDLCs were rinsed with PBS twice and fixed in 95% alcohol for 30 min, followed by PBS washes twice. The cells were stained with 0.2% ARS solution (Cyagen Biosciences, Guangzhou, China) at 37°C for 30 min and then rinsed with distilled water. Red mineralized nodules could be observed under the microscope (TS100, Nikon). The nodules were fully dissolved in 2% cetylpyridinium chloride (Sigma-Aldrich), and the absorbance at 562 nm was measured on the microplate reader to detect the relative content of calcified nodules (Zhao et al., 2020).

### Reverse Transcription Quantitative Polymerase Chain Reaction (RT-qPCR)

Total RNA of hPDLCs was extracted using TRIzol reagent (Invitrogen). RNA concentration and OD260/OD280 (1.8–2.0) were measured using a spectrophotometer (NanoVue™, General Electric, Boston, USA). Synthesis of cDNA (Takara, Otsu, Japan) and real-time PCR were performed using SYBR Green reagent (Bio Fact, Daejeon, South Korea). Relative expression of miR and mRNAs was calculated by 2^−ΔΔCt^ method, with U6 and glyceraldehyde-3-phosphate dehydrogenase (GAPDH) acting as the internal reference. The experiment was repeated three times. Primers (Table 1) were synthesized by Sangon Biotech (Shanghai, China).

**Table 1:** Primer sequence for RT-qPCR.

Name of primer	Sequences (5'-3')	Accession
KLF5-F	ATGGAGAAGTATCTGACACCTCA	NM_001286818
KLF5-R	TCAGTTCTGGTGCCTCTTCATATG	
RUNX2-F	ATGGCATCAAACAGCCTCTTCAGC	NM_001015051
RUNX2-R	TCAATATGGTCGCCAAACAGATTCA	
OCN-F	ATGAGAGCCCTCACACTCCTCG	NM_199173
OCN-R	CTAGACCGGGCCGTAGAAGCGCCG	
OPN-F	ATGAGAATTGCAGTGATTTGCT	NM_000582
OPN-R	TTAATTGACCTCAGAAGATGCACT	
ALP-F	ATGATTTCACCATTCTTAGTACTG	NM_000478
ALP-R	CAGAACAGGACGCTCAGGGGGTA	
miR-143-3p-F	TGAGATGAAGCACTG	MIMAT0000435
miR-143-3p-R	GAGCTACAGTGCTTC	
Wnt7b-F	ATGCACAGAAAC TTTCGCAAGT	NM_058238
Wnt7b-R	TCACTTGCAGGTGAAGACCTCG	
β-catenin-F	ATGGCTACTCAAGCTGATTTGA	NM_001098209
β-catenin-R	TTACAGGTCAGTATCAAACCAG	
U6-F	CGCTTCGGCAGCACATATAC	NR_004394
U6-R	AATATGGAACGCTTCACGA	
GAPDH-F	ATGGTTTACATGTTCCAATATGA	NM_001256799
GAPDH-R	TTACTCCTTGGAGGCCATGTGG	

### Western Blotting

The cells were lysed in pre-cooled radio-immunoprecipitation assay buffer (50 mM Tris–HCl, pH 7.5, 150 mM sodium chloride, 1% Triton X-100, 1% sodium deoxycholate, 2 mM ethylene diamine tetraacetic acid and 0.1% sodium dodecyl sulfate; Biosesang Inc., Sungnam, South Korea) containing protease inhibitor (Sigma-Aldrich). The lysate were centrifuged at 20,000 g for 20 min at 4°C to collect supernatant. The concentration of protein was tested using the bicinchoninic acid assay kit (Beyotime Biotechnology Co. Ltd., Shanghai, China). The lysis buffer (10 μg) was separated on 12% sodium dodecyl sulfate-polyacrylamide gel electrophoresis and transferred onto nitrocellulose membranes (Amersham Pharmacia Biotech, Piscataway, NJ, USA). Thereafter, the membranes were placed in the blocking buffer (P0023B, Beyotime) for 1 h, and cultured with the primary antibodies at 4°C overnight: β-actin (1/1000, ab8227, Abcam), KLF5 (1/1000, ab137676, Abcam), Wnt7b (1/10,000, ab155313, Abcam), Runx family transcription factor 2 (Runx2; 1/1000, ab236639, Abcam), osteocalcin (OCN; 1/1000, ab133612, Abcam), osteopontin (OPN; 1/1000, ab214050, Abcam), and β-catenin (1/1000, ab68183, Abcam). Afterwards, the membranes were incubated with the secondary antibody horseradish peroxidase-conjugated goat anti-rabbit immunoglobulin G (IgG) H&L (1/5000, ab205718, Abcam) for 2 h. Next, the membranes were developed and visualized using the enhanced chemiluminescence reagent (Merck Millipore, Billerica, MA, USA). The image of protein blotting was analyzed by ImageJ v1.48 software (National Institutes of Health, Bethesda, MD, USA) with β-actin as the internal reference. The experiment was repeated three times.

### Dual-Luciferase Reporter Gene Assay

The binding site of KLF5 and miR-143-3p was predicted by the database. The wild-type (WT) vector (KLF5-WT) and the mutant type (MUT) vector (KLF5-MUT; Ambion, Austin, TX, USA) were constructed. Then, 293T cells (American Type Culture Collection, Manassas, Virginia, USA) were seeded into the 24-well plates with 90% confluence. Next, 0.5 μg of KLF5-WT vector or KLF5-MUT vector was cotransfected with 40 nmol miR-143-3p mimic or 40 nmol mimic NC into 293 T cells using the Lipofectamine 2000 (Invitrogen). After 48 h, the cells were collected in the passive lysis buffer [a component of the dual-luciferase reporter gene detection system (Ambion, Austin, Texas, USA)]. Luciferase activity was tested using the GloMax®20/20 luminometer (Promega Corp., Madison, Wisconsin, USA).

### Statistical Analysis

Data analysis was introduced using the SPSS 21.0 (IBM Corp., Armonk, NY, USA). Kolmogorov-Smirnov method was used to check whether the data were in normal distribution. Data are expressed as mean ± standard deviation. One-way analysis of variance (ANOVA) or two-way ANOVA was used for the comparisons among multiple groups, followed by Sidak’s multiple comparisons test, Dunnett’s multiple comparisons test, or Tukey’s multiple comparisons test. The value of *p* was obtained from a two-tailed test, and *p* < 0.05 meant statistical significance.

## Results

### KLF5 Expression Was Enhanced During Osteogenic Differentiation of hPDLCs

**Figure 1:**
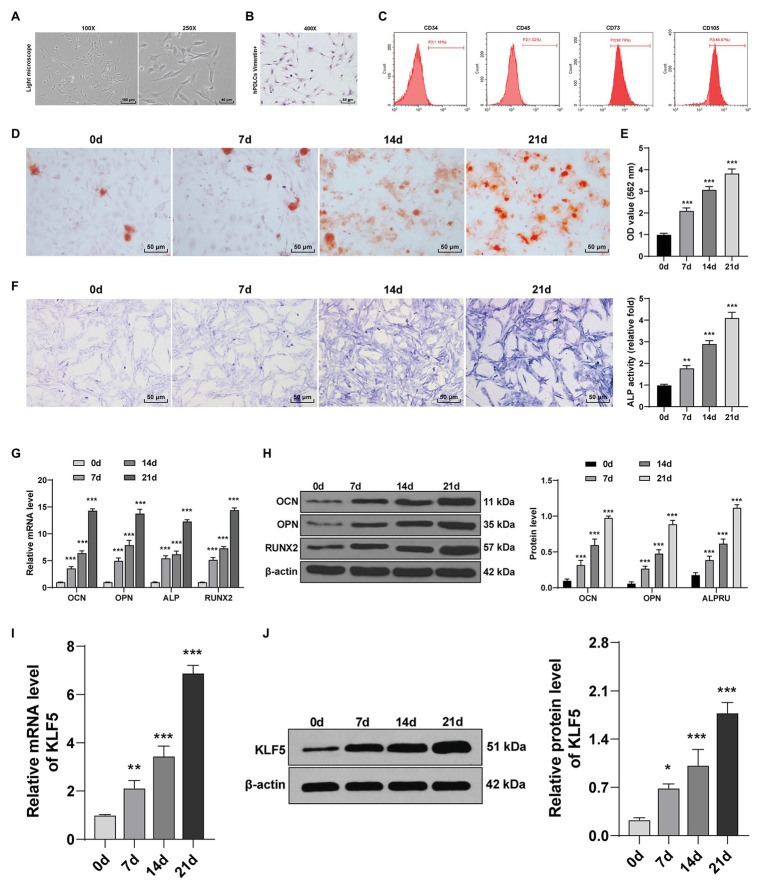
KLF5 expression was upregulated during osteogenic differentiation of hPDLCs. **(A)** Morphology of hPDLCs at passage 3. **(B)** Vimentin was measured using immunohistochemistry staining (400×, bar = 40 μm). **(C)** Expressions of surface markers of hPDLCs were identified using flow cytometry. **(D,E)** Osteogenic differentiation of hPDLCs was analyzed using ARS staining and ARS semi-quantitative analysis. **(F)** ALP activity was measured using ALP staining. **(G,H)** Levels of osteogenic specific markers (OCN, OPN, ALP, and Runx2) were detected using Reverse transcription quantitative polymerase chain reaction (RT-qPCR) and Western blotting. **(I,J)** KLF5 expression during the osteogenic differentiation of hPDLCs was detected using RT-qPCR and Western blotting. The experiment was repeated three times. Data are expressed as mean ± standard deviation. One-way ANOVA was applied to assess data in panels (**E**,**F**,**I**,**J**), and two-way ANOVA was applied to assess data in panels **(G,H)**, followed by Dunnett’s multiple comparisons test or Tukey’s multiple comparisons test, ^*^p < 0.05, ^**^p < 0.01, ^***^p < 0.001.

Osteogenesis of hPDLCs is crucial for periodontal tissue regeneration (Bian and Xiang, 2020). hPDLCs at passage 3 were spindle-shaped under the microscope (Figure 1A). Vimentin level was detected using immunohistochemistry, and we found that the cytoplasm was stained brown by anti-vimentin antibody (Figure 1B). Flow cytometry analysis showed that hPDLCs had MSC-related surface markers (CD73 and CD105) but did not express hematopoietic surface markers (CD34 and CD45; Figure 1C). It was indicated that hPDLCs were cultured successfully. Subsequently, osteogenic induction of hPDLCs was conducted. With the extension of induction time, the osteogenic differentiation ability of hPDLCs was increased gradually. ARS semi-quantitative analysis showed that the number of nodules was increased significantly (all *p* < 0.01; Figures 1D,E). Then, osteogenic specific markers (OCN, OPN, ALP, and Runx2) were detected. The results revealed that the levels of OCN, OPN, ALP, and Runx2 were promoted from the 7th to the 21st day (all *p* < 0.05; Figures 1F,G,H). KLF5 is differentially expressed during tooth development, and may play a vital role during in the osteogenesis of hPDLCs (Chen et al., 2009, 2017; Mihara et al., 2017). We exhibited that KLF5 expression was enhanced notably at the 7th, 14th, and 21st day after osteogenesis induction (all *p* < 0.05; Figures 1I,J). These results revealed that KLF5 might play a role during the osteogenesis of hPDLCs.

### KLF5 Silencing Inhibited the Osteogenic Differentiation of hPDLCs

**Figure 2:**
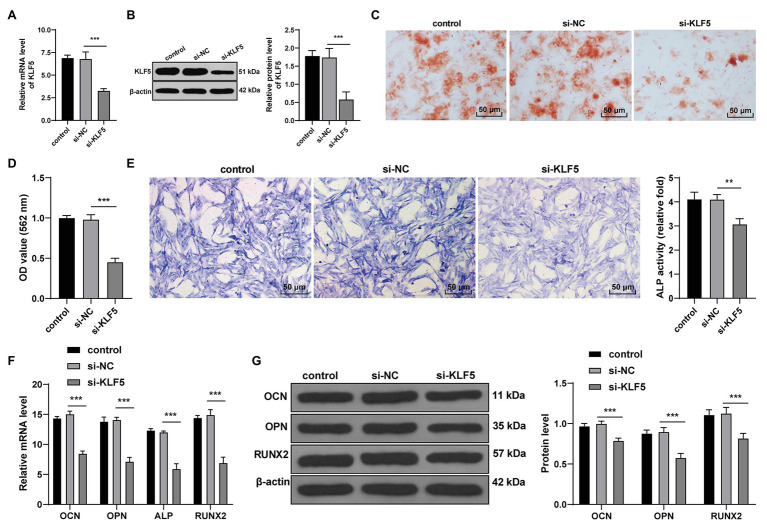
KLF5 silencing inhibited the osteogenic differentiation of hPDLCs. **(A,B)** Silencing effect of siRNA on KLF5 was measured using RT-qPCR and Western blotting. **(C,D)** Effect of KLF5 on osteogenic differentiation of hPDLCs was measured using ARS staining and ARS semi-quantitative analysis. **(E)** ALP activity was measured using ALP staining. **(F,G)** Changes of OCN, OPN, ALP, and Runx2 in hPDLCs were measured using RT-qPCR and Western blotting. Data are expressed as mean ± standard deviation. One-way ANOVA was applied to assess data in panels (**A**,**B**,**D**,**E**), and two-way ANOVA was applied to assess data in panels (**F**,**G**), followed by Dunnett’s multiple comparisons test or Tukey’s multiple comparisons test, ^**^p < 0.01, ^***^p < 0.001.

KLF5 was highly expressed during the osteogenic process of hPDLCs, and consequently, we speculated that KLF5 was related to the osteogenesis of hPDLCs. We transfected hPDLCs with si-KLF5, and the results of RT-qPCR and Western blotting confirmed the transfection efficiency of si-KLF5 (all *p* < 0.001; Figures 2A,B). Then, the effect of KLF5 on osteogenic differentiation of hPDLCs was detected. The results revealed that si-KLF5 attenuated the differentiation ability, reduced nodule number of hPDLCs (all *p* < 0.01; Figures 2C–E), and decreased the levels of OCN, OPN, ALP, and Runx2 in hPDLCs (all *p* < 0.001; Figures 2F,G). Taken together, KLF5 silencing restrained the osteogenesis of hPDLCs.

### miR-143-3p Targeted KLF5 in hPDLCs

**Figure 3:**
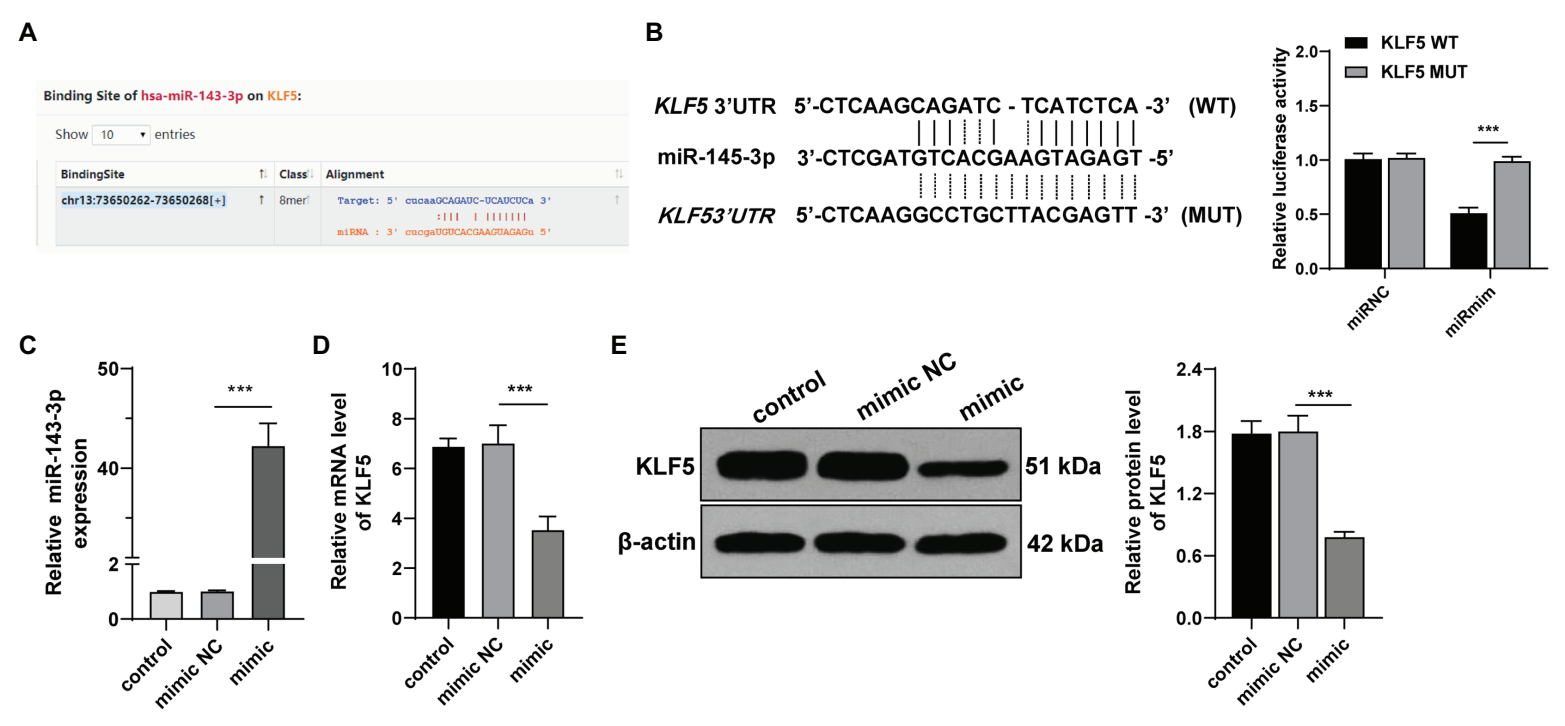
miR-143-3p targeted KLF5 in hPDLCs. **(A)** Binding site of miR-145-3p and KLF5 was predicted by Starbase. **(B)** Binding relationship between miR-145-3p and KLF5 was verified using dual-luciferase reporter gene assay. **(C)** Effect of miR-145-3p mimic on miR-145-3p was measured using RT-qPCR. **(D)** Effect of miR-145-3p mimic on mRNA level of KLF5 was measured using RT-qPCR. **(E)** Effect of miR-145-3p mimic on protein levels of KLF5 was measured using Western blotting. The experiment was repeated three times. Data are expressed as mean ± standard deviation. One-way ANOVA was applied to assess data in panel **(B)**, followed by Tukey’s multiple comparisons test, and two-way ANOVA was applied to assess data in panels **(C–E)**, followed by Sidak’s multiple comparisons test or Tukey’s multiple comparisons test, ^***^p < 0.001.

We then explored the molecular mechanism of KLF5 during osteogenesis of hPDLCs. miR negatively regulates target genes and affects cell differentiation by inducing mRNA degradation or inhibiting translation initiation (Beermann et al., 2016). The Starbase showed that KLF5 had targeting relationship with multiple miRs, among which miR-143-3p can restrain osteogenesis of human BMSCs (Wu et al., 2020). To determine whether miR-143-3p targeted KLF5 during osteogenic differentiation of hPDLCs, we performed dual-luciferase reporter gene assay according to the binding site of miR-143-3p and KLF5 (Figure 3A). It was confirmed that miR-143-3p targeted KLF5 (Figure 3B). Additionally, RT-qPCR showed that miR-143-3p mimic upregulated miR-143-3p expression in hPDLCs (*p* < 0.001; Figure 3C). miR-143-3p mimic reduced KLF5 expression during osteogenesis of hPDLCs (all *p* < 0.001; Figures 3D,E). These results suggested that miR-143-3p inhibited KLF5 expression in hPDLCs.

### miR-143-3p Targeted KLF5 to Inhibit Osteogenic Differentiation of hPDLCs

**Figure 4:**
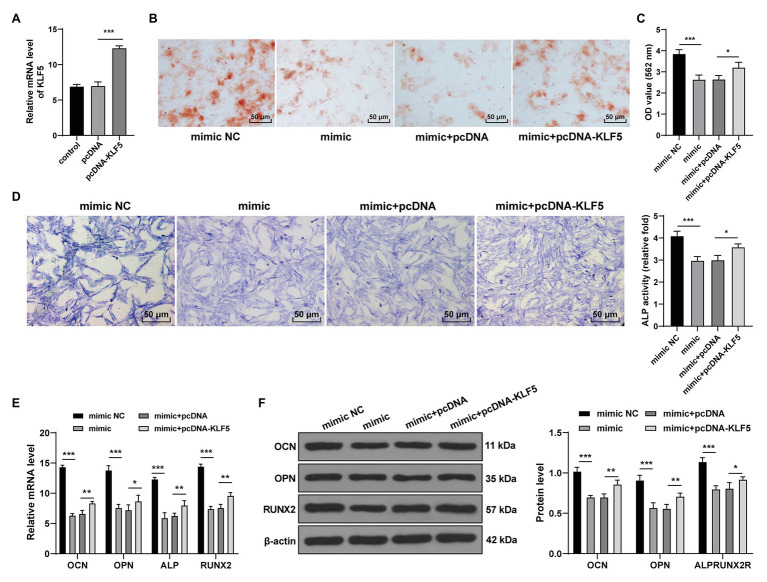
miR-143-3p targeted KLF5 to inhibit osteogenic differentiation of hPDLCs. **(A)** Effect of pcDNA-KLF5 on KLF5 expression was measured using RT-qPCR. **(B,C)** Osteogenic differentiation of hPDLCs was measured using ARS staining and ARS semi-quantitative analysis. **(D)** ALP activity was measured using ALP staining. **(E,F)** Levels of osteogenic specific markers (OCN, OPN, ALP, and Runx2) were detected using RT-qPCR and Western blotting. The experiment was repeated three times. Data are expressed as mean ± standard deviation. One-way ANOVA was applied to assess data in panels (**A**,**C**,**D**), and two-way ANOVA was applied to assess data in panels **(E,F)**, followed by Tukey’s multiple comparisons test, ^*^p < 0.05, ^**^p < 0.01, ^***^p < 0.001.

To verify the function of miR-143-3p/KLF5 during the osteogenesis of hPDLCs, we transfected hPDLCs with miR-143-3p mimic and pcDNA-KLF5, respectively, and then the effect of the interventions on osteogenic differentiation of hPDLCs was measured. RT-qPCR confirmed that the transfection of pcDNA-KLF5 was conducted successfully (*p* < 0.001; Figure 4A). The nodules and osteogenic ability of hPDLCs in the mimic group were higher than those in the mimic NC group, while the nodules and osteogenic ability of hPDLCs in the mimic + pcDNA-KLF5 group were lower than those in the mimic + pcDNA group (all *p* < 0.05; Figures 4B–D). miR-143-3p mimic decreased the levels of OCN, OPN, ALP, and Runx2, while the combined treatment increased the levels of OCN, OPN, ALP, and Runx2 (all *p* < 0.05; Figures 4E,F). Briefly, miR-143-3p mimic inhibited osteogenesis of hPDLCs, and overexpression of KLF5 could reverse such effect, indicating that miR-143-3p inhibited osteogenesis of hPDLCs by targeting KLF5.

### miR-143-3p/KLF5 Suppressed the Wnt/β-Catenin Pathway in hPDLCs

**Figure 5:**
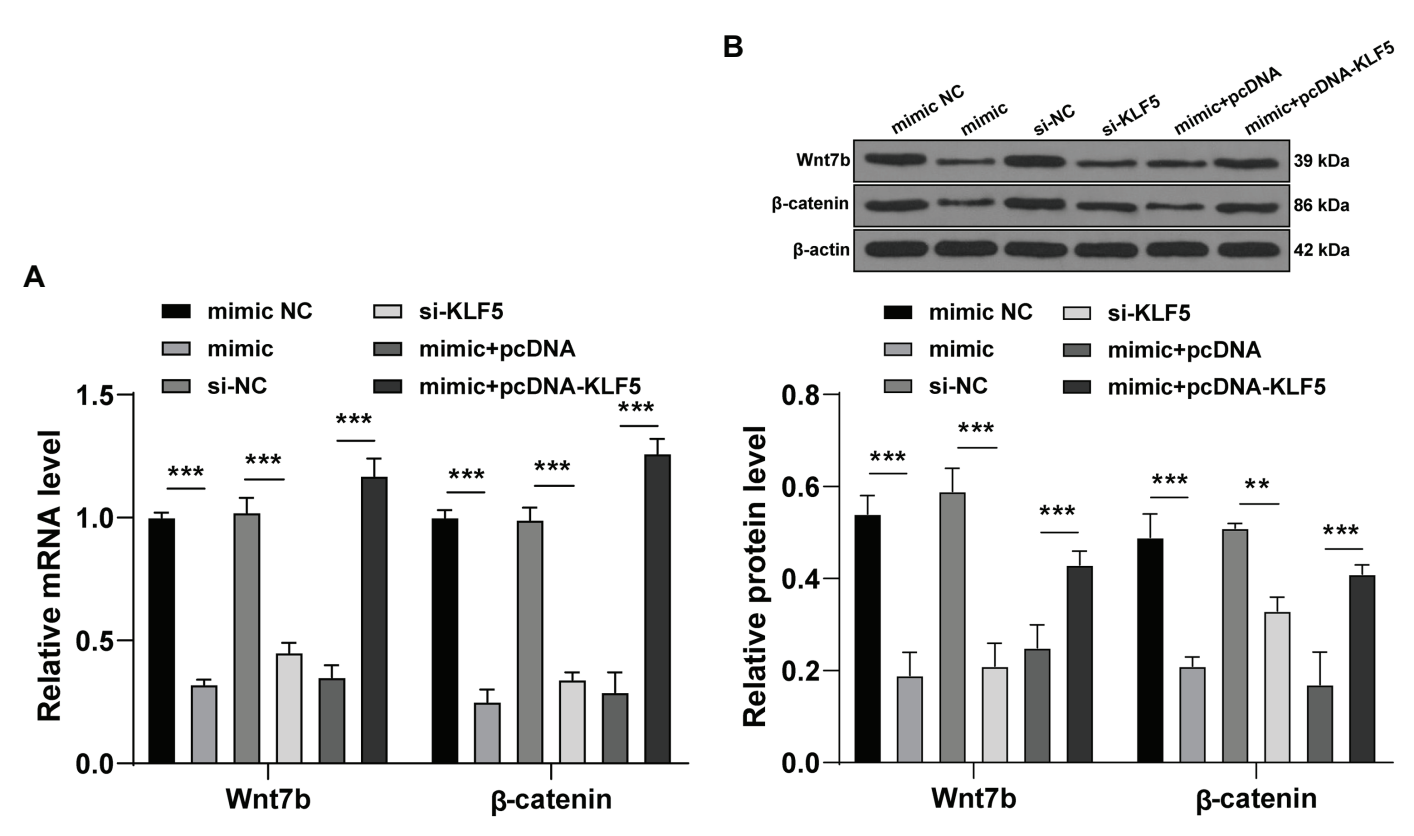
miR-143-3p targeted KLF5 and inhibited the Wnt/β-catenin pathway in hPDLCs. **(A,B)** After intervention of miR-143-3p and KLF5, protein levels of Wnt7b and β-catenin during osteogenic differentiation of hPDLCs were detected using RT-qPCR and Western blotting. The experiment was repeated three times. Data are expressed as mean ± standard deviation. Two-way ANOVA was applied to assess data in panels **(A,B)**, followed by Tukey’s multiple comparisons test, ^**^p < 0.01, ^***^p < 0.001.

Subsequently, we focused on the downstream mechanism of KLF5 during osteogenesis of hPDLCs. The key proteins of the Wnt/β-catenin pathway interact with KLF5 in many biological tissues (Guo et al., 2015), and the Wnt pathway relates to the osteogenesis of hPDLCs (Wang et al., 2020). Hence, we speculated that miR-143-3p/KLF5 modulated the osteogenesis of hPDLCs by affecting the Wnt/β-catenin pathway. After the intervention of miR-143-3p and KLF5 expression, the key factors of Wnt/β-catenin pathway during osteogenesis of hPDLCs were detected. The expressions of Wnt7b and β-catenin were declined after overexpression of miR-143-3p or inhibition of KLF5 (all *p* < 0.001; Figures 5A,B). The expressions of Wnt7b and β-catenin in hPDLCs in the mimic + pcDNA-KLF5 group were upregulated compared with those in the mimic + pcDNA group (all *p* < 0.001; Figures 5A,B). Briefly, miR-143-3p targeted KLF5 and restrained the Wnt/β-catenin pathway during osteogenesis of hPDLCs.

### Activation of the Wnt Pathway Reversed Repression Effect of miR-143-3p Mimic on Osteogenic Differentiation of hPDLCs

**Figure 6:**
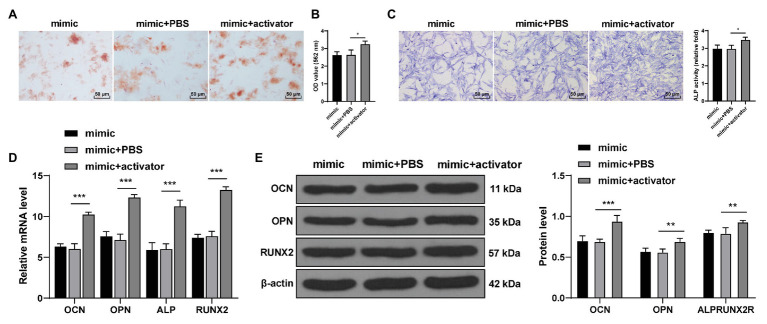
Activation of the Wnt pathway reversed the inhibitory effect of miR-143-3p mimic on osteogenic differentiation of hPDLCs. **(A,B)** Osteogenic differentiation of hPDLCs was measured using ARS staining and ARS semi-quantitative analysis. **(C)** ALP activity was measured using ALP staining. **(D,E)** Levels of osteogenic specific markers (OCN, OPN, ALP, and Runx2) were detected using RT-qPCR and Western blotting. The experiment was repeated three times. Data are expressed as mean ± standard deviation. One-way ANOVA was applied to assess data in panels (**B**,**C**), and two-way ANOVA was applied to assess data in panels **(D,E)**, followed by Dunnett’s multiple comparisons test or Tukey’s multiple comparisons test, ^*^p < 0.05, ^**^p < 0.01, ^***^p < 0.001.

Wnt pathway activator 2 was added to miR-143-3p mimic-treated hPDLCs to activate the Wnt/β-catenin pathway. The staining results showed that compared with the mimic + PBS-treated hPDLCs, the mimic + activator-treated hPDLCs showed increased mineralized nodules and enhanced osteogenic ability (all *p* < 0.05; Figures 6A–C). The levels of OCN, OPN, ALP, and Runx2 of hPDLCs in the mimic + activator group were notably higher than those in the mimic + PBS group (Figure 6B; all *p* < 0.01; Figures 6D,E). It was suggested that activating the Wnt/β-catenin pathway reversed repression effect of miR-143-3p mimic on osteogenesis of hPDLCs.

## Discussion

hPDLCs bear strong proliferation and differentiation potentials in periodontal tissues and consequently represent ideal candidates for periodontal tissue regeneration (Wu et al., 2017; Xue et al., 2018). KLF5 can stimulate osteogenic differentiation of hPDLCs under cyclic tensile stress (Gao et al., 2018). miRs contribute to MSC differentiation in bone tissue engineering by regulating osteogenic mineral deposition and marker gene expression (Carthew et al., 2020). miR-143 is demonstrated to be a suppressor of osteogenic differentiation (Li et al., 2014). This study revealed the repressive effect of miR-143-3p/KLF5 axis on osteogenic differentiation of hPDLCs.

The regeneration and repair of periodontal tissues mainly depend on the osteogenic differentiation ability of hPDLCs (Li et al., 2018). The role of KLF5 in odontoblast differentiation and dentin formation has been unveiled (Du and Hu, 2017). Han et al. have revealed that KLF5 expression is elevated during the odontoblastic differentiation of dental pulp cells *in vitro* (Han et al., 2017). We exhibited that KLF5 expression was increased notably during the osteogenesis of hPDLCs, and hPDLCs transfected with si-KLF5 showed decreased nodules and reduced osteogenic ability. OCN is viewed as a biomarker of bone mineralization (Neve et al., 2013), and OPN contributes to the bone remodeling, biomineralization, and periodontal regeneration (Singh et al., 2018). ALP represents a vital biomarker of osteogenic activity of hPDLCs (Jiang and Hua, 2016). Runx2 is a major marker of the osteoblast differentiation and chondrocyte maturation (Komori, 2018). Our results showed that si-KLF5 decreased the levels of OCN, OPN, ALP, and Runx2 in hPDLCs. Similarly, knockdown of KLF5 inhibits ALP activity of dental pulp cells during odontoblast differentiation (Han et al., 2017) and decreased Runx2 expression and calcification (Zhang et al., 2014). KLF5 enhances proliferation and osteogenic differentiation of hPDLCs under cyclic tensile stress (Gao et al., 2018). Overall, we exhibited that KLF5 silencing inhibited the osteogenic differentiation of hPDLCs.

Osteogenic differentiation is a developmental progression where cytokines, growth factors, signal transduction pathway and miRs interact together (Moghaddam and Neshati, 2019). We then focused on the miR that regulated KLF5 expression during osteogenic differentiation of hPDLCs. The Starbase showed that KLF5 had targeting relationships with multiple miRs, including miR-143-3p. miR-143-3p is closely concerned with the osteogenesis of adipose-derived stem cells (Jia et al., 2018). Downregulation of miR-143-5p is required for the promotion of odontoblasts differentiation of human dental pulp stem cells (hDPSCs; Wang et al., 2019a). In this study, the targeting relationship between miR-143-3p and KLF5 was verified using dual-luciferase reporter gene assay. miR-143-3p mimic inhibited the osteogenesis of hPDLCs and overexpression of KLF5 could reverse such effect. Consistently, miR-143-3p inhibition is demonstrated to elevate the expression of ALP, OCN, and OPN, and enhance mineralization and hDPSC apoptosis (Yang et al., 2020). Briefly, we were the first to exhibit that miR-143-3p targeted KLF to suppress osteogenesis of hPDLCs.

Thereafter, we shifted to exploring the downstream molecular mechanism of KLF5 during osteogenic differentiation of hPDLCs. The Wnt/β-catenin pathway can exert critical effects on tooth development, morphological changes and dental cell differentiation (Jarvinen et al., 2018). Importantly, the Wnt/β-catenin pathway is implicated in the progression of osteogenic differentiation of hPDLCs (Zheng et al., 2019). There is a certain interaction between KLF5 and the Wnt/β-catenin pathway (Guo et al., 2015). We speculated that miR-143-3p/KLF5 monitored osteogenesis of hPDLCs by affecting the Wnt/β-catenin pathway. Our results exhibited that the levels of Wnt7b and β-Catenin were suppressed after overexpression of miR-143-3p or inhibition of KLF5. Activating the Wnt pathway reversed the inhibitory effect of miR-143-3p mimic on osteogenesis of hPDLCs. Correspondingly, Wang et al. (2020) have also revealed that increasing level of nuclear β-catenin can promote osteogenesis of hPDLCs. Transfection of β-catenin shRNA restrains levels of osteoblast-related genes including ALP, Runx2, OCN, and OPN (Zhang et al., 2018). Taken together, these results indicated that miR-143-3p/KLF5 suppressed the Wnt/β-catenin pathway during osteogenesis of hPDLCs.

To sum up, miR-143-3p restrains osteogenesis of hPDLCs by targeting KLF5 and inactivating the Wnt/β-catenin pathway. The novelty of this study lies in the discovery that the miR-143-3p/KLF5 axis plays a role in osteogenic differentiation of hPDLCs *via* the Wnt/β-catenin signaling pathway. There are also some limitations of this study. The in-depth mechanism of the miR-143-3p/KLF5 axis in the Wnt/β-catenin pathway has not been fully clarified. Additionally, whether the miR-143-3p/KLF5 axis can be employed as a breakthrough point for tooth treatment needs further study. In the future, we shall determine the mechanism of miR-143-3p/KLF5 axis in Wnt/β-catenin from the perspective of epigenetics.

## Data Availability Statement

The original contributions presented in the study are included in the article/supplementary material, further inquiries can be directed to the corresponding author.

## Ethics Statement

The studies involving human participants were reviewed and approved by the Clinical Ethical Committee of Affiliated Hospital of Hainan Medical University. Written informed consent to participate in this study was provided by the participants’ legal guardian/next of kin. The animal study was reviewed and approved by the Clinical Ethical Committee of Affiliated Hospital of Hainan Medical University.

## Author Contributions

KW, CH, and ZLa made substantial contributions to the conception of the present study. ZLu, WF, CLh, and CLo performed the experiments and wrote the manuscript. ZLi and YT contributed to the design of the present study and interpreted the data. All authors read and approved the final version of the manuscript for publication.

### Conflict of Interest

The authors declare that the research was conducted in the absence of any commercial or financial relationships that could be construed as a potential conflict of interest.
